# On Connected Target Coverage for Wireless Heterogeneous Sensor Networks with Multiple Sensing Units

**DOI:** 10.3390/s90705173

**Published:** 2009-06-30

**Authors:** Kuei-Ping Shih, Der-Jiunn Deng, Ruay-Shiung Chang, Hung-Chang Chen

**Affiliations:** 1 Department of Computer Science & Information Engineering, Tamkang University, Tamshui 251, Taipei, Taiwan; E-Mail: kpshih@mail.tku.edu.tw; 2 Department of Computer Science & Information Engineering, National Changhua University of Education, Changhua, Taiwan; E-Mail: djdeng@cc.ncue.edu.tw; 3 Department of Computer Science & Information Engineering, National Dong Hwa University, Hualien, Taiwan; 4 Department of Information Technology, Ching Kuo Institute of Management and Health, Keelung, Taiwan; E-Mail: gileschen@ems.cku.edu.tw

**Keywords:** connectivity, heterogeneous sensors, multiple sensing units, target coverage, wireless sensor networks

## Abstract

The paper considers the connected target coverage (CTC) problem in wireless heterogeneous sensor networks (WHSNs) with multiple sensing units, termed MU-CTC problem. MU-CTC problem can be reduced to a connected set cover problem and further formulated as an integer linear programming (ILP) problem. However, the ILP problem is an NP-complete problem. Therefore, two distributed heuristic schemes, REFS (remaining energy first scheme) and EEFS (energy efficiency first scheme), are proposed. In REFS, each sensor considers its remaining energy and its neighbors’ decisions to enable its sensing units and communication unit such that all targets can be covered for the required attributes and the sensed data can be delivered to the sink. The advantages of REFS are its simplicity and reduced communication overhead. However, to utilize sensors’ energy efficiently, EEFS is proposed. A sensor in EEFS considers its contribution to the coverage and the connectivity to make a better decision. To our best knowledge, this paper is the first to consider target coverage and connectivity jointly for WHSNs with multiple sensing units. Simulation results show that REFS and EEFS can both prolong the network lifetime effectively. EEFS outperforms REFS in network lifetime, but REFS is simpler.

## 1. Introduction

With the advancements of Micro-Electro-Mechanical System (MEMS), digital electronics, as well as wireless communication technology, a kind of small-size, low-cost, and low-power device with sensing, processing and wireless transmission capabilities, called *sensor*, is widely developed and deployed in a variety of applications. A wireless sensor network (WSN) is an auto-configured network consisted of many sensors deployed in a sensing field in an ad hoc or prearranged fashion. The purposes of WSNs include sensing, monitoring, or tracking environmental events. WSNs have been widely used in battlefield surveillance, environmental monitoring, biological detection, home automation, industrial diagnostics, etc. [1].

A wireless heterogeneous sensor network (WHSN) is a sub-class of wireless sensor networks in which each sensor may have different capabilities, such as various transmission capabilities, different number of sensing units, etc. [2, 3]. In the paper, a WHSN with multiple sensing units is considered, which means each sensor in the WHSN may be equipped with more than one sensing unit, and the *attribute* that each sensing unit can sense may also be different. In fact, sensors equipped with multiple sensing units are very common in many commercial products. For example, each MICA2 mote [4] is equipped with several sensing units for temperature, humidity, light, sound, vibration, etc. A WHSN with multiple sensing units is inherently formed in nature because some sensing units in a sensor may be malfunctioned after running for a long time. The remaining sensing units on each sensor may be different. As a result, how to utilize the sensors with the remaining sensing units efficiently to continue the original sensing task is a very important concern.

Furthermore, using a WHSN with multiple sensing units is also cost-effective and power-efficient if multiple attributes are required to be sensed in the sensing field. On one hand, in addition to the sensing unit, a sensor, in general, consists of a control unit, a power unit, a radio unit, etc. If a sensor is equipped with only one sensing unit, it will increase the cost substantially to deploy all kinds of sensors to sense all required attributes. On the other hand, if too many sensing units are equipped in a sensor, the sensor will quickly run out of energy. Therefore, a WHSN with multiple sensing units is a promising deployment if multiple attributes are required to be sensed in the sensing field [2, 3]. Moreover, it is very likely that several different kinds of sensors have been deployed in the sensing field for different purposes. These sensors can collaborate for additional sensing purposes to increase the sensor utilization.

*Coverage* and *connectivity* are two key factors to a successful WSN. In general, coverage problems either deploy sensors to cover the sensing field completely [5, 6], or make sure that all the sensing field is covered by a certain amount of sensors, such as 1-coverage or *k*-coverage [7, 8], or select active sensors in a densely deployed sensor networks to cover all the sensing field [9–12]. On the other hand, the connectivity issue emphasizes how well sensors connect to the sink and if the sensed data can be properly delivered to the sink. The connected target coverage (CTC) problem is one of the target coverage (TC) problems, but also takes the connectivity issue into consideration simultaneously. In this paper, CTC problem in a WHSN with multiple sensing units is termed MU-CTC problem (where MU means multiple sensing units) and is defined as below.

**Definition 1 (MU-CTC Problem)** *Given a set of targets (or points) of interest and a number of sensors with multiple sensing units randomly deployed in the sensing field, MU-CTC problem is to schedule the on/off of the sensing units as well as the communication unit on each sensor such that (1) the attributes required to be sensed at each target can be sensed at all time, (2) the sensed data can be delivered to the sink, and (3) the network lifetime is maximized.*

The network lifetime is defined as the time interval from the beginning to the time that either the condition (1) or (2) above is not satisfied.

MU-CTC problem can be represented by a bipartite graph and be reduced to a connected set cover problem, named MU-CSC (**M**ultiple sensing **U**nits for **C**onnected **S**et **C**over) problem. The MU-CSC problem can be formulated as an integer linear programming (ILP) problem and solved by an ILP solver. However, solving the ILP problem is NP-complete [13]. Therefore, two distributed schemes, named REFS (remaining energy first scheme) and EEFS (energy efficiency first scheme), are proposed to deal with the MU-CTC problem. In REFS, a sensor enables its sensing and communication units based on its remaining energy and its neighbors’ decisions. The advantages of REFS are its simplicity and reduced communication overhead. However, redundant sensing is the most significant weakness of REFS.

Generally in the CTC problem, a sensor not only undertakes the sensing task, but also needs to relay the sensed data for others. Therefore, to make the best use of a sensor’s energy, target coverage and sensed data relay should be considered simultaneously. Consequently, EEFS is proposed, where a sensor enables its sensing and communication units by considering not only its target coverage but also its relay role. As a result, the network lifetime of EEFS can be prolonged accordingly. Simulation results also verify that EEFS outperforms REFS in network lifetime. In addition, to our best knowledge, this is the first paper to discuss such a problem in the literature.

The rest of the paper is organized as follows. Section 2 describes the related work that treated the CTC problem with different network models and assumptions. Section 3 formulates MU-CTC problem as an ILP problem. Section 4, two distributed schemes, REFS and EEFS, are proposed to deal with the MU-CTC problem. Simulation results are presented in Section 5. Section 6 concludes the paper.

## 2. Related Work

Coverage problem is of critical importance for wireless sensor networks. As described above, the coverage problem either deploys sensors to cover the sensing field, or selects active sensors and schedule them to cover all the sensing field, or analyzes how well the sensing field is covered. Recently, the target coverage problem has received extensive attention [13–20]. Nevertheless, none of them considered sensors equipped with multiple sensing units. Moreover, some of them do not take the connectivity issue into account. These researches are summarized as follows.

In [13], the authors transformed the TC problem into a *Maximal Set Cover* (MSC) problem, where sensors are organized into set covers. To maximize the number of set covers is equivalent to maximizing the network lifetime. Every set cover is activated in turn and the sensors in an activated set cover are responsible for sensing all the targets at a specific time, while all the other sensors are in the sleep state. The MSC problem was proved to be an NP-complete problem and two heuristics were proposed to solve the MSC problem using linear programming and greedy techniques, respectively. However, these schemes are centralized. Similar to [13] except for fixed sensing range, the TC problem with adjustable sensing range is addressed in [14], where the goals of the problem are to schedule sensors to alternate between the active and the sleep states and adjust their sensing ranges so that all targets are covered by active sensors and the network lifetime is maximized. The authors transformed the problem to an *adjustable range set covers* (AR-SC) problem and formulated it by ILP constraints. The authors then solved it using relaxation and rounding techniques. A greedy heuristic is proposed, where both centralized and distributed (localized) solutions are given for computing the set covers. However, connectivity is not considered in [13, 14].

In [15], the TC problem is considered. The proposed approach consists the following three steps. A linear programming method is used to compute the maximum network lifetime. A workload matrix was used to specify the total length of time that a sensor should watch a target. The workload matrix is further decomposed into a sequence of schedule matrices by using the perfect matching method. Finally, the target watching timetable is obtained for each sensor based on the schedule matrices. A similar approach is also used in [16, 17], where the connectivity issue is jointly considered with the TC problem. In [16], *k*-coverage is additionally taken into account. That is, given *k*, it requires each target to be covered by at least *k* sensors and those active sensors to be connected. However, the proposed approaches in [15–17] are centralized. Moreover, they also assume that a sensor can only monitor at most one target at a time, which simplifies the difficulty in dealing with the problem.

Similarly, the CTC problem is also considered in [18]. The authors model the CTC problem as a Maximum Cover Tree (MCT) problem, where the sensors in a cover tree can cover all the targets and relay the sensed data to the sink. Based on the MCT problem, the upper bound of the CTC problem in terms of lifetime is derived by using a linear programming model. The Communication Weighted Greedy Cover (CWGC) algorithm is further proposed to construct the cover trees. However, the CWGC algorithm is centralized and practically hard to implement.

In [19], the CTC problem with *k*-coverage is considered. Two non-global schemes, cluster-based and pruning-based, are proposed in the paper. The cluster-based scheme selects the backbone sensors to form a *k*-connected coverage set. In the pruning-based scheme, each sensor determines its status (marked or unmarked) based on its two-hop neighborhood information. All marked sensors form a *k*-connected coverage set. In [20], the CTC problem in a WHSN is considered, where the WHSN means that the network consists of two types of sensors. One is the resource-rich sensors called supernodes used for data relaying and the other is the energy constrained sensors. In the paper, supernodes are assumed to have two transceivers, one for communication with sensors and the other for communication with other supernodes. All supernodes form a connected network and the active sensor connects to at least one supernode, via either a direct or a multi-hop connection. The problem is transformed to a heterogeneous connected set covers (HCSC) problem and the HCSC problem is proved to be NP-complete. An ILP approach as well as a distributed and localized approach are proposed in the paper.

Table 1 briefly summarizes the related work. Although there are a lot of related work in the literature that deal with TC or CTC problem, all of the above works [13–20] only considered each sensor to be equipped with one sensing unit. As described earlier, a WHSN with multiple sensing units is a very common, useful and important. Thus, the TC or CTC problem deserve receiving more attention toward a WHSN with multiple sensing units. In our previous research [21], the TC problem in a WHSN with multiple sensing units was investigated, but the connectivity issue was not taken into account. This paper now discusses the CTC problem in a WHSN with multiple sensing units. The CTC problem aiming at heterogeneous sensors with multiple sensing units turns out to be more complicated than those focusing on homogeneous sensors with only one sensing unit. The reasons are as follows. In WHSNs with multiple sensing units, the CTC problem needs to consider not only which sensors need to be activated, but also which sensing units on those sensors need to be activated. Moreover, the sensing attributes that need to be covered at each target are different. In addition, the connectivity issue is also considered in the paper. In [13], the maximal set cover problem considering a sensor with single sensing unit has been proven to be an NP-complete problem. The CTC problem in WHSNs with multiple sensing units is a superset of that with only one sensing unit. Thus, the CTC problem in a WHSN with multiple sensing units is also an NP-complete problem.

As a result, two heuristic schemes are proposed in the paper to schedule the sensors’ sensing units as well as the communication unit such that the individually required attributes of a given set of targets can be covered all the time, the sensed data can be relayed to the sink, and the network lifetime can be maximized.

## 3. Problem Statements and Formulations

### 3.1. Assumptions

In a sensing field *𝒜*, there are *M* stationary targets at known locations to be continuously sensed. Let *t_m_* denote the *m*th target, *m* = 1, 2, …, *M*. Suppose there are *L* attributes, denoted *a^l^, l* = 1, 2, …, *L*, to be sensed for these targets. The attributes to be sensed on each target are not altogether the same. Attribute *a^l^* can be sensed by the sensing unit *u^l^*, *l* = 1, 2, …, *L*. The energy consumption of the sensing unit *u^l^* in sensing attribute *a^l^* for a time unit is *e^l^*, *l* = 1, 2, …, *L*. Suppose there are *N* stationary sensors equipped with different numbers of sensing units randomly deployed in the sensing field. Let *s_n_* denote the *n*th sensor, *n* = 1, 2, …, *N*. The initial energy of each sensor is *E* and assumed the same. All the sensed data are required to be forwarded to the sink. Without loss of generality, let *s*_0_ denote the sink for easy description.

In this paper, each sensor knows its location and can obtain one-hop neighbor information via communication. Moreover, the locations of the targets to be sensed are known by the sensors in advance and will not change during the whole sensing period. In reality, the sensing ranges of different kinds of sensing should be different. However, the sensing range of each sensing unit as well as the communication range of each sensor are all assumed to be the same and unadjustable. The assumption can be easily relaxed as follows. Knowing the equipped sensing units as well as the location information of its one-hop neighbors and itself, each sensor can determine the attributes that can be sensed by itself or by its one-hop neighbors via communication. Therefore, the proposed protocols are expected to work well even when the assumption is relaxed.

Let *R_s_* denote the sensing range of a sensing unit and *R_c_* the communication range of a sensor. For simplicity, the paper also assumes that *R_c_* ≥ 2*R_s_*. It is to make sure that two sensors can communicate directly if their sensing ranges overlap. If $R_{s}>\frac{1}{2}R_{c}$, for any sensor, the information of whether a required sensing attribute at a target can be sensed by its neighbors has to be collected through multi-hop communication. However, how to acquire the information through multi-hop communication is not discussed here and will be of future work.

Target *t_m_* is said to be monitored by *u^l^* of *s_n_*, if *d*(*t_m_*, *s_n_*) ≤ *R_s_* and *s_n_* is equipped with *u^l^*, where *d*(*x, y*) is the Euclidean distance between *x* and *y*. Two sensors, say *s_n_* and *s*_*n*′_, can are communicate if *d*(*s_n_*, *s*_*n*′_) ≤ *R_c_*. Namely, they are neighbors.

**Figure 1.**
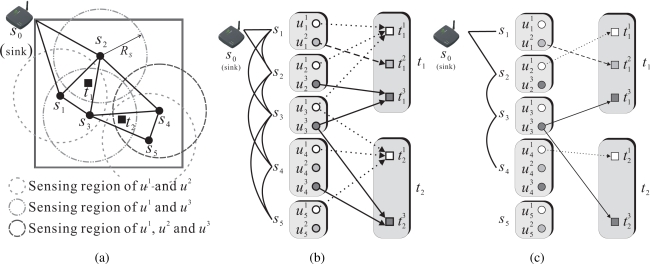
An illustrated example, where five sensors are scheduled to cover two targets. (a) The topology. (b) The coverage and connectivity relationships. (c) An illustration of a connected set cover.

The MU-CTC problem can be represented by a quasi-bipartite graph and be further formulated as a connected set cover problem, named **M**ultiple Sensing **U**nits for **C**onnected **S**et **C**over (MU-CSC) problem. Take Figure 1 as an example, where five sensors, *s*_1_, *s*_2_, …, *s*_5_, are required to sense two targets, *t*_1_ and *t*_2_. The attributes required to be sensed at *t*_1_ are *a*^1^, *a*^2^, and *a*^3^ and those at *t*_2_ are *a*^1^ and *a*^3^. As shown in Figure 1(a), *t*_1_ is located within the sensing ranges of *s*_1_, *s*_2_, and *s*_3_, and *t*_2_ is within the sensing ranges of *s*_3_, *s*_4_, and *s*_5_. The sensing units equipped by *s*_1_, *s*_2_, *s*_3_, *s*_4_, and *s*_5_ are {*u*^1^, *u*^2^}, {*u*^1^, *u*^3^}, {*u*^1^, *u*^3^}, {*u*^1^, *u*^2^, *u*^3^}, and {*u*^1^, *u*^2^}, respectively. Figure 1(a) illustrates the network topology, where different circles represent the different sensing capabilities of sensors and the black solid lines represent the connectivity among sensors.

### 3.2. The MU-CSC Problem

Let $u_{n}^{l}$ denote the sensing unit *u^l^* on sensor *s_n_*, if sensor *s_n_* is equipped with sensing unit *u^l^*, for *n* = 1, 2, …, *N* and *l* = 1, 2, …, *L*. Moreover, let $t_{m}^{l}$ stand for the attribute *a^l^* required to be sensed at target *t_m_*, *m* = 1, 2, …, *M* and *l* = 1, 2, …, *L*. The MU-CTC problem can be represented by a quasi-bipartite graph, as illustrated in Figure 1(b). There exists a ray from $u_{n}^{l}$ to $t_{m}^{l}$ if the sensing unit *u^l^* on sensor *s_n_* can sense the attribute *a^l^* at target *t_m_*. In Figure 1, the different types of rays mean different types of sensing units. In addition, the black solid lines between two sensors mean that these sensors can communicate with each other directly.

Consequently, the MU-CTC problem can be regarded as a maximal connected set cover problem. Sensors are organized as connected set covers. In each connected set cover, the sensors turn on some sensing units to cover the required attributes of the targets, or turn on their communication units to relay the sensed data to the sink. Figure 1(c) is an example of a connected set cover for the example shown in Figure 1(a). The set $\left\{u_{1}^{2},\;u_{2}^{1},\;u_{3}^{3},\;u_{4}^{1}\right\}$ is a connected set cover, where $u_{1}^{2}$ covers $t_{1}^{2}$, $u_{2}^{1}$ covers $t_{1}^{1}$, $u_{3}^{3}$ covers $t_{1}^{3}$ and $t_{2}^{3}$, and $u_{4}^{1}$ covers $t_{2}^{1}$. In addition, *s*_1_, *s*_2_, *s*_3_, and *s*_4_ can relay the sensed data to the sink via multi-hop transmission. The MU-CSC problem is defined as follows.

**Definition 2 (MU-CSC Problem)** *Given M targets and N sensors with multiple sensing units, the MUCSC problem is to find a family of connected set covers C*_1_, *C*_2_, ..., *C_κ_*, *so that (1) the required attributes at every target can be completely covered by each connected set cover, (2) all sensors in a connected set cover can connect to the sink directly or via the sensors in the same connected set cover, (3) the energy consumption of each sensor in all connected set covers is at most E, the initial energy, (4) κ is maximized.*

Notice that, each connected set cover corresponds to a working period, say a *round* (to be described later). Thus, maximizing *κ* is equal to maximizing the network lifetime.

### 3.3. ILP Constraints for the MU-CSC Problem

In modeling ILP, some assumptions are made to simplify the formulations. Assume that a sensing unit on an active sensor periodically generates a sensed data in a round, where an active sensor is the sensor selected to undertake the sensing task. Data aggregation is not considered in the model. That is, if a sensor receives *f* sensed data, it needs to relay the *f* data to the sink, or transmit *f* +1 data out if its data is counted. Without loss of generality, the index variables listed below are used for their corresponding meanings, if not otherwise specified.

- *m: m*th target, where 1 ≤ *m* ≤ *M*,
- *n: n*th sensor, where 1 ≤ *n* ≤ *N*, and
- *l: l*th attributes, sensing unit, or energy consumption of the *l*th sensing unit, where 1 ≤ *l* ≤ *L*.

By Definition 2, the MU-CSC problem can be modeled by the ILP constraints as follows.

**ILP Constraints for the MU-CSC Problem**

*Given:*

- *M* targets: *t_m_*, *m* = 1, 2, …, *M*,
- *N* sensors: *s_n_*, *n* = 1, 2, …, *N*,
- The sink: *s*_0_,
- Initial energy of each sensor: *E*,
- *L* sensing attributes: *a^l^*, *l* = 1, 2, …, *L*,
- *L* sensing units: *u^l^*, which respectively senses the attribute *a^l^*, and the energy consumed is: *e^l^*, *l* = 1, 2, …, *L*,
- *e_t_*: the energy consumption of a sensor to transmit a sensed data,
- *e_r_*: the energy consumption of a sensor to receive a data,
- ℵ (*n*): the neighbor set of *s_n_* and is defined as {*n*′|*d*(*s_n_*, *s*_*n*′_) ≤ *R_c_*, *n*′ = 0, 1, …, *N, n*′ ≠ *n*}, where *d*(*s_n_*, *s*_*n*′_) is the Euclidean distance between *s_n_* and *s*_*n*′_,
- $v_{n}^{l}$: a coefficient that indicates whether *s_n_* is equipped with *u^l^*; $v_{n}^{l}=1$ if *s_n_* is equipped with *u^l^* ; otherwise, $v_{n}^{l}=0$, *n* = 1, 2, …, *N*, and *l* = 1, 2, …, *L*,
- *μ_n,m_*: a coefficient that indicates whether *s_n_* can cover *t_m_*; *μ_n,m_* = 1 if *d*(*s_n_*, *t_m_*) ≤ *R_s_*; otherwise, *μ_n,m_* = 0, *n* = 1, 2, …, *N*, and *m* = 1, 2, …, *M*,
- $\tau_{m}^{l}$: a coefficient that indicates whether *t_m_* needs to be covered by *a^l^*; $\tau_{m}^{l}=1$ if *t_m_* needs to be covered by *a^l^*; otherwise, $\tau_{m}^{l}=0$, *m* = 1, 2, …, *M*, and *l* = 1, 2, …, *L*.

*Variables:*

- *c_k_*: a boolean variable; *c_k_* = 1 if *C_k_* is a connected set cover; otherwise, *c_k_* = 0, ∀ *k* = 1, 2, …, *K*, where *K* is an upper bound of the number of connected set covers,
- ${\hat{\nu }}_{n,k}^{l}$: a boolean variable; ${\hat{\nu }}_{n,k}^{l}=1$ if sensor *s_n_* enables the sensing unit *u^l^* in set *C_k_*; otherwise, ${\hat{\nu }}_{n,k}^{l}=0$, ∀ *n, l, k*,
- $f_{n\to n^{\prime }}^{k}$: a non-negative integer that indicates the number of sensed data relayed from *s_n_* to *s*′*_n_* in set *C_k_*.

*Objective*: Maximize *c*_1_ + *c*_2_ + … + *c*_K_.

*Subject to*:

(**C1**) $\sum_{n=1}^{N}\left(\mu_{n,m}*\nu_{n}^{l}*{\hat{\nu }}_{n,k}^{l}\right)\geq c_{k}*\tau_{m}^{l},\forall m,\;l,\;k,$
(**C2**) $\sum_{k=1}^{K}\;\left(\sum_{l=1}^{L}\;\left({\hat{\nu }}_{n,k}^{l}*e^{l}\right)+e_{t}\sum_{n^{\prime }\in ℵ(n)}\;f_{n\to n^{\prime }}^{k}+e_{r}\;\sum_{n^{\prime }\in ℵ(n)}\;f_{n^{\prime }\to n}^{k}\right)\leq E,\;\forall \;n,$
(**C3**) $\sum_{n^{\prime }\in ℵ(n)}\;f_{n\to n^{\prime }}^{k}-\sum_{n^{\prime }\in ℵ(n)}\;f_{n^{\prime }\to n}^{k}=\sum_{l=1}^{L}\;{\hat{\nu }}_{n,k}^{l},\;\forall n,\;k,$
(**C4**) $\sum_{n\in ℵ(0)}\;f_{n\to 0}^{k}=\sum_{n=1}^{N}\;\sum_{l=1}^{L}\;{\hat{\nu }}_{n,k}^{l},\;\forall k,$
(**C5**) ${\hat{\nu }}_{n,k}^{l}\leq c_{k},\;\forall \;n,\;l,\;k,$
(**C6**) $0\leq f_{n\to n^{\prime }}^{k}\leq N*L,\;\forall \;n,\;n^{\prime },\;k,$
(**C7**) ${\hat{\nu }}_{n,k}^{l}\in \{0,1\},\;\forall \;n,\;l,\;k,$
(**C8**) *c_k_* ∈ {0, 1}, ∀ *k*.

Remarks:

- Constraint **C1** corresponds to the first requirement in Definition 2. The constraint guarantees that the required attributes at every target can be completely covered by any selected connected set cover.
- Constraint **C2** corresponds to the third requirement in Definition 2. The constraint indicates that the total energy consumption of a sensor should not exceed its initial energy *E*.
- Constraint **C3** is to ensure that the generated and the received data of a sensor in a round can be forwarded by its neighbors.
- Constraint **C4** is used to make sure that the amount of the sensed data generated by the sensors belonging to a connected set cover is equal to that received by the sink in a round. Therefore, with constraints **C3** and **C4**, it can be ensured that the sensed data of a sensor can be relayed to the sink, that is, the second requirement in Definition 2.
- Constraint **C5** is to require a sensor to not to turn on its sensing units if the sensor does not belong to a connected set cover. It helps to further reduce the energy consumption of a sensor.
- Constraints **C6-C8** indicate the ranges of these variables.

Note that, it is well-known that solving ILP is an NP-complete problem[13]. Therefore, two distributed schemes to solve the MU-CTC problem are proposed.

## 4. Distributed Schemes for the MU-CTC Problem

**Figure 2.**
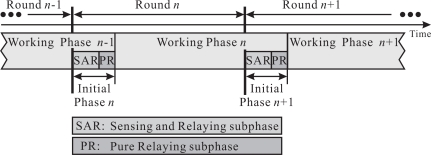
Time structure.

It is well-known that solving ILP is an NP-complete problem. Therefore, two distributed schemes, named REFS (remaining energy first scheme) and EEFS (energy efficiency first scheme), are proposed to solve the MU-CTC problem. As shown in Figure 2, time is divided into rounds of equal length. A round consists of an *initial phase* and a *working phase*. The initial phase is further divided into a *sensing and relaying (SAR)* subphase and a *pure relaying (PR)* subphase. Let *𝒟_Init_*, *𝒟_SAR_*, and *𝒟_PR_* denote the durations of the initial phase, the SAR subphase and the PR subphase, respectively. Clearly, *𝒟_Init_* = *𝒟_SAR_* + *𝒟_PR_*. Note that *𝒟_Init_* is much shorter than the duration of a round.

During *𝒟_SAR_*, each sensor will determine which of its sensing units should be turned on. If the sensor needs to turn on some sensing units, it still needs to find an appropriate neighbor to relay the sensed data. Similarly, the relay node also needs to find its relay node to continue relaying the sensed data to the sink. Basically, whether a sensor needs to be activated and which of its sensing units need to be turned on will be decided during *𝒟_SAR_*. Consequently, *𝒟_PR_* is only for the sensor chosen as the relay to find its relay to the sink. The working phase begins at the end of the initial phase and ends at the end of the initial phase of the next round so that the targets can be continually monitored. In addition, in both REFS and EEFS, each sensor makes the decision only by one-hop neighbor information, such as the location, the remaining energy, and the sensing capability of neighbors, as well as the requests for relaying.

### 4.1. A Generic Approach to the MU-CTC Problem

Algorithm 1 is a generic approach to the MU-CTC problem, which is performed by each sensor during every initial phase in a distributed fashion. Initially, in Step 1, each sensor will set a waiting time, say *W_n_* for sensor *s_n_*, in order to receive the neighbors’s decision and then make its own decision after *W_n_* expires. It is worth mentioning that *W_n_* heavily impacts the performance of the proposed schemes. As a result, in designing *W_n_*, REFS takes the sensor’s remaining energy into account so that the sensor with more remaining energy can make the decision sooner to take the coverage burden. On the other hand, in EEFS, both the coverage and connectivity are taken into account to efficiently utilize the sensing and communication units of its neighbors’ and its own.

**Algorithm 1:** A generic approach to the MU-CTC problem

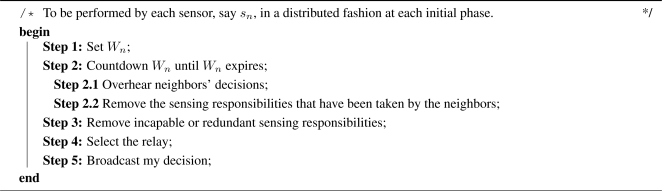

In Step 2, each sensor counts down *W_n_* and waits for the neighbors’ decision until *W_n_* expires. After *W_n_* expires, the sensor can decide which sensing and communication units need to be turned on. Therefore, in Step 3, some strategies are adopted to remove the redundant sensing responsibilities or to make the sensing responsibilities more efficiently performed. In Step 4, the relay selection is performed to find an appropriate sensor to relay the sensed data. Finally, in Step 5, the decision, including which sensing units need to be turned on, whether the communication unit needs to be turned on, and which neighbor is selected as the relay, is announced to its neighbors. Based on the generic approach shown in Algorithm 1, the following two subsections describe the two distributed algorithms, REFS and EEFS, in detail. Notice that the proposed algorithms do not require all sensor to be accurately synchronized. Each sensor only needs to be synchronized with its neighbors. The local synchronization can be achieved through the decision announcement in Step 5.

### 4.2. Remaining Energy First Scheme (REFS)

REFS is a self-pruning approach, which takes the sensor’s remaining energy and neighbors’ decisions into account to enable its sensing and communication units. Based on Algorithm 1, the details of REFS are illustrated as follows.

#### Set *W_n_*

Let $\delta_{n}^{l}$ be the sensing capability of the sensing unit *u^l^* on sensor *s_n_* and be defined as $\delta_{n}^{l}=\left\{t_{m}^{l}|\mu_{n,m}*\nu_{n}^{l}*\tau_{m}^{l}=1,\;\forall \;m\right\}$. Furthermore, let Δ*_n_* be the sensing capability of sensor *s_n_*, which is the union of the sensing capabilities of all sensing units equipped on *s_n_*. That is, $\Delta_{n}=\cup_{\forall \nu_{n}^{l}=1}\delta_{n}^{l}$. For the example shown in Figure 1, $\delta_{1}^{2}=\left\{t_{1}^{2}\right\}$ and $\delta_{3}^{1}=\left\{t_{1}^{1},t_{2}^{1}\right\}$. In addition, $\Delta_{1}=\delta_{1}^{1}\cup \delta_{1}^{2}=\left\{t_{1}^{1},t_{1}^{2}\right\}$ and $\Delta_{3}=\delta_{3}^{1}\cup \delta_{3}^{3}=\left\{t_{1}^{1},\;t_{2}^{1},\;t_{1}^{3},\;t_{2}^{3}\right\}$. Initially, a sensor, say *s_n_*, will take Δ*_n_* as its sensing responsibility. Let Γ*_n_* denote the *sensing responsibility* of *s_n_*. Thus, Γ*_n_* is initialized to Δ*_n_*.

In REFS, the setting of *W_n_* solely depends on the remaining energy of the sensor. The more the energy remains, the shorter is the waiting time. As a result, the sensor with more energy will turn on more sensing units to sense the attributes of the targets. Let $E_{n}^{r}$ stand for the remaining energy of *s_n_*. *W_n_* is set as follows:

$$W_{n}=\left(1-\frac{E_{n}^{r}}{E}\right)*{𝒟}_{\mathrm{SAR}}.$$

#### Countdown *W_n_*

At the beginning of every initial phase, each sensor, say *s_n_*, will wait for a waiting time of its own (*W_n_*) and overhear the decisions of the neighbors with smaller waiting time. While receiving the neighbors’ decision packets, the sensor will prune away from Γ*_n_* those $t_{m}^{l}$ indicated in the neighbors’ decision packets. Note that, in the neighbors’ decision packets, the next relay is also included. If *s_n_* is indicated as a relay, *s_n_* should turn on its communication unit and also need to find the next relay for itself in order to relay the sensed data for the neighbors.

#### Remove incapable sensing responsibilities

After *W_n_* expires, the remaining Γ*_n_* is the sensing responsibility of *s_n_* at this round. However, it is possible that all remaining energy of *s_n_* is still not enough to support all sensing units indicated in Γ*_n_*. As a result, *s_n_* will orderly remove the sensing units whose sensing capability is the least. If *S* is a set, |*S*| means the cardinality of *S*. Therefore, the deletion of Γ*_n_* can be formally represented as follows.

$$\Gamma_{n}=\Gamma_{n}-\left\{t_{m}^{l^{\prime }},\;\forall \;m|l^{\prime }=\text{arg}\;\underset{l\in L}{\text{min}}|\left\{t_{m}^{l}\in \Gamma_{n},\;\forall \;m\right\}|\right\}.$$

Finally, *s_n_* enables the corresponding sensing units indicated in the remaining Γ*_n_* to cover the attributes for the indicated targets.

#### Select the relay

Upon deciding to turn on the sensing units or being indicated as other’s relay, *s_n_* will select a neighbor *s*_*n*′_ to relay the sensed data to the sink. In general, the sensors closest to the sink are the best. Let *nextRelay*(*n*) denote the relay sensor for *s_n_*. Therefore, in REFS, *nextRelay*(*n*) is set as below.

$$\mathrm{nextRelay}(n)=\text{arg}\;\underset{n^{\prime }\in ℵ(n)}{\text{min}}\;d(s_{n^{\prime }},\;s_{0}).$$

Note that the way to find the relay is the same as geographic routing. However, in geographic routing, a local minimum (dead end) problem will occur. Many approaches have been proposed to handle the problem [22, 23]. Therefore, the paper does not address the problem. In addition, if *s_n_* does not have enough energy to enable any sensing unit, it will quit executing REFS and turn off all sensing units. The remaining energy of *s_n_* will be left for communication only.

#### Broadcast my decision

Finally, *s_n_* announces its decision as well as the selected relay.

#### Summary

Overall, REFS is a simple scheme that can be easily implemented. Moreover, REFS incurs less control overhead. However, REFS only takes the sensor’s remaining energy and the neighbors’ decisions into account for making its own decision. The degree of contributions of the sensing units and the communication unit are not considered in REFS. Therefore, the sensor’s energy can not be efficiently utilized. As a result, the sensor applying REFS has a high possibility to enable more redundant sensing or communication units. Consequently, the network lifetime can not be prolonged effectively.

### 4.3. Energy Efficiency First Scheme (EEFS)

Similar to REFS, EEFS is also a self-pruning approach and is operated at every initial phase by each sensor to individually schedule the on/off state of the sensing and communication units for the following working phase. However, in addition to remaining energy, EEFS also takes coverage and connectivity into consideration. EEFS adds more heuristics to prune the redundant or inefficient sensing responsibilities away and select the relay intelligently.

Before executing EEFS, each sensor has to collect its neighbors’ sensing capabilities and critical sensing responsibilities in advance in order to make the most efficient use of its sensing and communication units. The *critical sensing responsibility* of a sensor is the attributes of the targets (in terms of $t_{m}^{l}$) that can be sensed only by that sensor. Let Θ*_n_* be the critical sensing responsibility of *s_n_*. $\Theta_{n}=\left\{t_{m}^{l}|\mu_{n,m}*\nu_{n}^{l}*\tau_{m}^{l}=1\;\text{and}\;\sum_{n^{\prime }\in ℵ(n)}\;\nu_{n^{\prime }}^{l}=0,\;\forall l,\;m\right\}$. For the example shown in Figure 1, $\Theta_{1}=\left\{t_{1}^{2}\right\}$. In other words, each sensor, say *s_n_*, has to compute Δ*_n_* and Θ*_n_* as well as collect Δ_*n*′_ and Θ_*n*′_, *∀* *n*′ ∈ ℵ(*n*), in advance. Nevertheless, the collection needs to be performed only once. Moreover, to balance the energy consumption and efficiently utilize the energy of sensors, each sensor has to exchange $E_{n}^{r}$ with its neighbors at the beginning of EEFS. Based on Algorithm 1, the details of EEFS are described as follows.

#### Set *W_n_*

In EEFS, sensor *s_n_* calculates *W_n_* by the following information: (1) the sensing capabilities, (2) the critical sensing responsibilities, (3) the remaining energy of itself and its neighbors, as well as (4) the probability of being others’ relay. According to the sensing capabilities as well as the critical sensing responsibilities of itself and its neighbors, sensor *s_n_* will rank its sensing priority among its neighbors. The sensor with higher sensing priority and lower probability of being others’ relay has shorter *W_n_* so that the sensor can make its decision sooner. A sensor’s sensing priority and the probability of being others’ relay are regarded as its *coverage contribution* and *connectivity contribution*, respectively.

#### Coverage contribution

The coverage contribution of a sensor reflects the ranking of the sensor among its neighbors in regard to the sensing capability. The ranking is processed as follows. If *s_n_* is equipped with the sensing unit *u^l^*, *s_n_* will sort the sensing capability of $\delta_{n^{\prime }}^{l}$ in an increasing order to a list *𝒧^l^* according to $\left|\delta_{n^{\prime }}^{l}\right|,\;\forall \;n^{\prime }\;\in \left\{n\right\}\;\cup \;ℵ(n)$. If the sensing capabilities of two sensors are the same, the higher priority will be assigned to the one with more remaining energy. Otherwise, the sensor with a larger ID wins. Notice that only the sensor equipped with *u^l^* is included in the ranking process. Let $r_{n}^{l}$ be the order in *𝒧^l^*, which represents the priority of the sensing unit *u^l^* of *s_n_* among its neighbors. The larger the $r_{n}^{l}$ is, the higher its priority is. It is worth mentioning that the reason to take a rank among the neighbors is to normalize the $\left|\delta_{n^{\prime }}^{l}\right|$ for different *u^l^* on *s_n_*.

Take *s*_1_ in Figure 1 as an example. Since *ℵ*(*s*_1_) = {*s*_2_, *s*_3_}, only *s*_1_, *s*_2_, and *s*_3_ are taken into account. Suppose *E* = 8, $E_{1}^{r}=6$, $E_{2}^{r}=8$, and $E_{3}^{r}=4$. Since *s*_1_ is equipped with *u*^1^ and *u*^2^, $r_{1}^{1}$ and $r_{1}^{2}$ as well as $r_{\max}^{1}$ and $r_{\max}^{2}$ are to be calculated, where $r_{\max}^{l}=\left|{𝒧}^{l}\right|$. Firstly, *u*^1^ is considered. Obviously, $\left|\delta_{1}^{1}\right|=1$, $\left|\delta_{2}^{1}\right|=1$, and $\left|\delta_{3}^{1}\right|=2$. Since $\left|\delta_{1}^{1}\right|=\left|\delta_{2}^{1}\right|=1$, the remaining energy is taken into account. Therefore, the priority of $\delta_{1}^{1}$ among *s*_1_ and its neighbors is: $\delta_{1}^{1}<\delta_{2}^{1}<\delta_{3}^{1}$. That is, ${𝒧}^{1}=(\delta_{1}^{1},\;\delta_{2}^{1},\;\delta_{3}^{1})$. Consequently, $r_{1}^{1}=1$ and $r_{\max}^{1}=3$. With regard to *u*^2^, since *s*_2_ and *s*_3_ are not equipped with *u*^2^, therefore, $r_{1}^{2}=1$ and $r_{\max}^{2}=1$.

Let *ρ_n_* be the priority of *s_n_*, which takes the priorities of all sensing units equipped on *s_n_* into consideration. *ρ_n_* is set as:

$$\rho_{n}=\sum_{l{|}_{\nu_{n}^{l}=1}}\;\frac{r_{n}^{l}}{r_{\max}^{l}}.$$

For the above example, $\rho_{1}=\frac{1}{3}+\frac{1}{1}=\frac{4}{3}$.

Because the number of sensing units equipped on sensors is different, the coverage contribution of *s_n_*, denoted *ρ̄_n_*, is defined as the average priority of the sensing units equipped on *s_n_* and represented as follows.

(1) $${\bar{\rho }}_{n}=\frac{\rho_{n}}{\sum_{l{|}_{\nu_{n}^{l}=1}}\;\nu_{n}^{l}}=\frac{\sum_{l{|}_{\nu_{n}^{l}=1}}\;\frac{r_{n}^{l}}{r_{\max}^{l}}}{\sum_{l{|}_{\nu_{n}^{l}=1}}\;\nu_{n}^{l}}.$$

Notice that 0 < *ρ̄_n_* ≤ 1. With regard to the example in Figure 1, ${\bar{\rho }}_{1}=\frac{4}{3}/2=\frac{2}{3}$.

#### Connectivity contribution

The connectivity contribution of a sensor is defined as the probability of being others’ relay. Let *σ_n_* be the connectivity contribution of *s_n_*. *σ_n_* is determined as follows.

**Figure 3.**
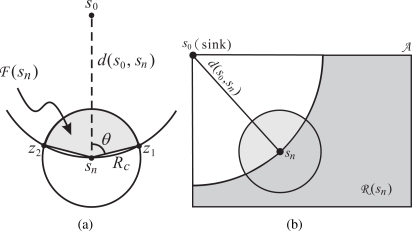
(a) The forwarding zone of *s_n_*, *𝒡* (*s_n_*). (b) The relaying zone of *s_n_*, *𝒭*(*s_n_*).

It is hard for a sensor to find the best route to the sink merely by its local information, such as the one-hop neighbor information. Like REFS, the neighbor with the shortest distance to the sink is the best candidate to relay the sensed data for the sensor. Therefore, a sensor closest to the sink has a higher probability to serve as others’ relay. Based on the concept, the location of the relay for sensor *s_n_* can only be located in the *forwarding zone* of *s_n_*. The definition of the forwarding zone of *s_n_* is defined as follows, where *C*(*s, R*) denotes a circle centered at *s* with the radius *R*. Figure 3 (a) also illustrates the forwarding zone of *s_n_*.

**Definition 3 (Forwarding Zone)** *Let the intersection points of two circles C*(*s*_0_, *d*(*s*_0_, *s_n_*)) *and C*(*s_n_*, *R_c_*) be *z*_1_ *and z*_2_*. The forwarding zone of s_n_*, *denoted 𝒡*(*s_n_*), *is defined as the circular sector formed by two radii* $\bar{s_{n}z_{1}}$ *and* $\bar{s_{n}z_{2}}$, *and the arc* $\hat{z_{1}z_{2}}$.

Compared with *s_n_*, the sensor located in the forwarding zone of *s_n_* has a shorter to-sink-distance, which is defined as the distance from the sensor to the sink. On the contrary, the sensor located in the area *C*(*s_n_*, *R_c_*) *− 𝒡*(*s_n_*) has a longer to-sink-distance than *s_n_*. It implies that the sensor located in *C*(*s_n_*, *R_c_*) *− 𝒡*(*s_n_*) may choose *s_n_* as its relay. Consequently, the more the sensors are located in *C*(*s_n_*, *R_c_*) *− 𝒡*(*s_n_*), the more likely *s_n_* become others’ relay. Therefore,

$$\sigma_{n}=\frac{\left|\left\{s_{n^{\prime }}|s_{n^{\prime }}\;\in \;\left(C\left(s_{n},\;R_{c}\right)-𝒡\left(s_{n}\right)\right)\right\}\right|}{\left|ℵ(n)\right|}.$$

The higher the *σ_n_* is, the more likely *s_n_* become others’ relay. Similarly, consider the same example in Figure 1. Because *s*_2_ and the sink, regarded as a neighbor of *s*_1_, are located at 𝒡(*s*_1_), $\sigma_{1}=\frac{3-2}{3}=\frac{1}{3}$.

#### The setting of *W_n_*

A sensor with a higher coverage contribution can more efficiently cover the targets, that is, consume less energy but cover more targets. Therefore, the sensor with a higher coverage contribution shall make its decision sooner, implying a shorter *W_n_*. On the other hand, a sensor with a higher connectivity contribution has a higher probability to relay. If the sensor is selected as a relay, a longer waiting time can let it make its decision later, to be likely to turn on its sensing units to cover the targets, and balance its energy consumption. Consequently, the sensor with a higher connectivity contribution shall wait for a longer time to make its decision.

As a result, a sensor with a higher coverage contribution and a lower connectivity contribution will have a shorter *W_n_* so that the sensor can determine whether it should turn on its sensing and/or communication units. *W_n_* is set as follows.

(2) $$W_{n}=1-\left(\alpha_{n}\;*\;\left(1-\sigma_{n}\right)+\left(1-\alpha_{n}\right)*\;{\bar{\rho }}_{n}\right)]*{𝒟}_{\mathrm{SAR}},$$

where *α_n_* is the *contribution tendency* of *s_n_* on coverage contribution or connectivity contribution. Basically, a sensor near the sink may have a higher probability to forward others the sensed data, because all the sensed data should be forwarded to the sink. The sensor near the sink shall have a higher connectivity tendency to relay the sensed data in order to keep the network connected. On the other hand, a sensor covering more targets shall have a higher coverage contribution tendency to sense the targets. Therefore, each sensor in the sensing field shall have different *α_n_* to make the best use of its contribution tendency. In other words, in addition to the status of neighbors, the hardware difference and the location of a sensor should be considered to determine *W_n_*. How to determine each sensors’ *α_n_* will be shown in detail in next subsection.

In order to demonstrate how the coverage and connectivity contributions affects *W_n_*, assume that the *α_n_* of the sensors in the example in Figure 1 are the same and set to 0.5. That is, the locations and the energy model of the sensors in Figure 1 is assumed similar. According to Equation (2), $W_{1}=\left[1-\left(\alpha_{1}*\left(1-\sigma_{1}\right)+\left(1-\alpha_{1}\right)*{\bar{\rho }}_{1}\right)\right]*{𝒟}_{\mathrm{SAR}}=\left[1-\left(0.5*\left(1-\frac{1}{3}\right)+\left(1-0.5\right)*\frac{2}{3}\right)\right]*{𝒟}_{\mathrm{SAR}}=\frac{1}{3}{𝒟}_{\mathrm{SAR}}$. Similarly, *W*_2_ can be calculated as follows. Assume $E_{4}^{r}=7$, ${\bar{\rho }}_{2}=\frac{8}{3}/2=\frac{3}{4}$. On the other hand, because only the sink is located in 𝒡(*s*_2_), $\sigma_{2}=\frac{4-1}{4}=\frac{3}{4}$. Therefore, $W_{2}=\left[1-\left(0.5*\left(1-\frac{3}{4}\right)+\left(1-0.5\right)*\frac{3}{4}\right)\right]*{𝒟}_{\mathrm{SAR}}=\frac{1}{2}{𝒟}_{\mathrm{SAR}}$. Clearly, *s*_1_ will make its decision first, because *W*_1_ < *W*_2_. In Figure 1, both *s*_1_ and *s*_2_ are equipped with two kinds of sensing units that can sense the corresponding sensing attributes at *t*_1_. Therefore, *s*_1_ and *s*_2_ have approximately the same coverage contribution. However, because the number of *s*_2_’s neighbors is more than that of *s*_1_, *s*_2_ should make its decision later to ensure the network connectivity.

#### The setting of *α_n_*

The tendency of a sensor toward coverage or connectivity is determined according to the locations of the targets, which are known by all sensors in advance. If the amount of sensed data to be relayed by a sensor is large, the sensor will take a higher priority in relaying the sensed data, instead of sensing the targets. Therefore, a *relaying zone* of a sensor is defined to estimate the amount of sensed data to be relayed by the sensor. Let *𝒭*(*s_n_*) denote the *relaying zone* of *s_n_*. Definition 4 and Figure 3(b) give the formal definition and an illustration of *𝒭*(*s_n_*), respectively.

**Definition 4 (Relaying Zone)** *Let 𝒭*(*s_n_*) *denote the relaying zone of s_n_*.

$$𝒭(s_{n})=\left(𝒜-C\left(s_{0},\;d\left(s_{0},\;s_{n}\right)\right)\right)\cup C(s_{n},\;R_{c}).$$

It is possible that the sensed data from the targets located in *𝒭*(*s_n_*) may need *s_n_* to relay to the sink. Therefore, the number of targets located in *𝒭*(*s_n_*) can be regarded as the amount of sensed data which needs *s_n_* to relay to the sink. As a result, a sensor with a larger number of targets in *𝒭*(*s_n_*) shall have a higher connectivity contribution tendency. Formally, *α_n_* of *s_n_* is set as follows.

$$\alpha_{n}=ɛ*\frac{\left|\left\{t_{m}|t_{m}\;\in \;𝒭(s_{n})\right\}\right|}{M},$$

where ɛ is a system adjustable parameter to reflect the hardware difference. If the energy cost of the communication unit is much higher than that of the sensing unit, even if the sensor has a higher priority in coverage contribution, the sensor should still pay more attention on the connectivity contribution. In contrast, if the energy cost of the sensing unit is higher than that of the communication unit, the sensor should put a higher weight on coverage contribution, regardless of the distance from the sensor to the sink. Therefore, ɛ is set high when the energy cost of the sensing unit is lower than that of the communication unit. Otherwise, ɛ is set low accordingly. Basically, ɛ is set between 0 and 1.

Notice that *α_n_* is based on the hardware characteristic, e.g., the energy consumption models of sensing and communication units, and the environment characteristic, e.g., the locations of sensors. These characteristics have an influence on whether a sensor can spend its energy efficiently on sensing or communication. However, these characteristics of a sensor are unchanged after the sensor is deployed. Therefore, it is not sufficient for the sensor to efficiently use its energy. When determining *W_n_*, a sensor also has to locally take the coverage and connectivity contributions among its neighbors and itself, as well as the remaining energy into consideration to meet the sensing requirements, to ensure the network connectivity, and to prolong the network lifetime.

#### Countdown *W_n_*

The processes to be performed here are the same as those in Step 2 of REFS.

#### Remove incapable or redundant sensing responsibilities

Upon the expiration of *W_n_*, the remaining Γ*_n_* is the sensing responsibility of *s_n_* in this round. However, it is still possible for *s_n_* to alleviate its burden via pruning out redundant sensing responsibilities. For example, for some neighbor of *s_n_*, say *s*_*n*′_, if Θ_*n*′_ ≠ ∅, *s*_*n*′_ has the responsibility to cover the sensing responsibilities indicated in Θ_*n*′_. Suppose *s*_*n*′_ turns on *u^l^*, for some *l*, to cover $t_{m}^{l}\left(\in \;\Theta_{n^{\prime }}\right)$, for some *m*. If turning on the sensing unit *u^l^* also covers the other targets, say $t_{m^{\prime }}^{l}$, for some *m*′ (that is, $t_{m^{\prime }}^{l}\;\in \;\delta_{n^{\prime }}^{l}$) and $t_{m^{\prime }}^{l}\;\in \;\Gamma_{n}$, therefore, $t_{m^{\prime }}^{l}$ can be pruned awat from Γ*_n_*. As a result, Γ*_n_* can be further improved.

In addition, it is possible to improve by pruning the inefficient sensing responsibilities of *s_n_* if it is better to leave these responsibilities to the neighbors with higher sensing efficiency. As defined above, $\delta_{n}^{l}$ is the sensing capability of $u_{n}^{l}$. The more $\left|\delta_{n}^{l}\right|$ is, the more targets $u_{n}^{l}$ can cover at a time. Therefore, $\left|\delta_{n}^{l}\right|$ can be regarded as the *benefit* of $u_{n}^{l}$ if $u_{n}^{l}$ is turned on. On the contrary, $\frac{e^{l}}{E_{n}^{r}}$ can be regarded as the *cost* of $u_{n}^{l}$, where *e^l^* and $E_{n}^{r}$ are the energy consumption of *u^l^* in sensing for a time unit and the remaining energy of *s_n_*, respectively. The cost considers not only the energy consumption of *u^l^*, but also takes the remaining energy of *s_n_* into account in order to reflect the effect of the energy consumption of *u^l^* on the remaining energy of *s_n_*. Consequently, $\frac{\left|\delta_{n}^{l}\right|}{e^{l}/E_{n}^{r}}$ can be regarded as the *benefit-cost ratio* (BCR) of $u_{n}^{l}$. In addition to the BCR of $u_{n}^{l}$, the sensing efficiency of $u_{n}^{l}$ on *s_n_* should take the ratio of the remaining energy of *s_n_* to the initial energy into consideration as well. Therefore, let $\mathrm{BCR}(u_{n}^{l})$ and *Eff*(*s_n_*, *u^l^*) denote the BCR of $u_{n}^{l}$ and the sensing efficiency of *u^l^* on *s_n_*, respectively. Accordingly, the sensing efficiency of *u^l^* on *s_n_*, *Eff*(*s_n_*, *u^l^*), is defined as below.

(3) $$\mathrm{Eff}(s_{n},\;u^{l})=(1-\alpha_{n})*\;\mathrm{BCR}\left(u_{n}^{l}\right)*\;\frac{E_{n}^{r}}{E},\;\;\text{where}\;\;\mathrm{BCR}\left(u_{n}^{l}\right)=\frac{\left|\delta_{n}^{l}\right|}{\frac{e^{l}}{E_{n}^{r}}}.$$

In designing *Eff*(*s_n_*, *u^l^*), the contribution tendency is also considered to differentiate the sensors nearby or far away from the sink with the same sensing unit to increase the sensing efficiency. To do so can further alleviate the sensing responsibility of *s_n_*. As a result, if there exists a sensor, say *s*_*n*′_, who has not sent out the **DecAnn** packet and whose sensing efficiency of *u^l^* is better than that of *u^l^* on *s_n_*, then *s_n_* will leave the sensing responsibilities covered by *u^l^* to *s*_*n*′_.

#### Select the relay

In EEFS, in addition to the neighbor’s to-sink-distance, the remaining energy of the neighbor is also taken into account for *s_n_* to select its relay. Basically, the neighbor with more remaining energy and shorter to-sink-distance will be selected as a relay. However, it is possible that the neighbor with a longer to-sink-distance may be selected. This will result in a longer path to the sink. Therefore, in EEFS, the neighbors located in *𝒡*(*s_n_*) are considered as relay candidates, instead of all the neighbors. Formally, *nextRelay*(*n*) is set as below.

$$\mathrm{nextRelay}(n)=\text{arg}\;\underset{n^{\prime }\in 𝒡(s_{n})}{\text{min}}\frac{d\left(s_{n^{\prime }},s_{0}\right)}{{\text{max}}_{j\in 𝒡(s_{n})}\;d(s_{j},\;s_{0})}*\left(1-\frac{E_{n^{\prime }}^{r}}{E}\right),$$

where $\frac{d\left(s_{n^{\prime }},\;s_{0}\right)}{{\text{max}}_{j\in 𝒡(s_{n})}\;d\left(s_{j},\;s_{0}\right)}$ and $\frac{E_{n^{\prime }}^{r}}{E}$ are to normalize the to-sink-distance and the remaining energy of *s*_*n*′_, respectively. Notice that the case of |*𝒡*(*s_n_*)|= 0 is regarded as the local minimum problem and is not addressed in the paper.

#### Broadcast my decision

*s_n_* announces its decision by sending out a **DecAnn** packet.

#### Summary

As mentioned above, REFS has a significant drawback that a sensor may turn on too many redundant sensing units. It is because a sensor in REFS only considers its remaining energy and its neighbors’ decisions. However, EEFS can alleviate such a situation and make the best use of the sensing and communication units equipped on a sensor. In addition, the coverage and connectivity contributions are introduced in designing *W_n_* of *s_n_*. To do so can let the sensor make its decision sooner if it has a higher sensing capability and a lower probability to relay for others. The order of making a decision has a significant impact on the performance of both REFS and EEFS. Moreover, in EEFS, both the coverage and the connectivity contributions are taken into account. As a result, a sensor with a better coverage or connectivity contribution can make its best decision either in covering the target or in relaying the sensed data. Consequently, the network lifetime can be prolonged efficiently.

The complete REFS and EEFS algorithms are omitted due to the space limitation.

## 5. Performance Evaluations

In this section, scheduling multiple sensing units on each sensor to sense a given number of targets and relay the sensed data to the sink in a WHSN are simulated extensively. The simulation setting for the MU-CTC problem is summarized in Table 2. The numbers of sensors and targets are specified in each simulation. The number and types of sensing units on sensors are also specified in each simulation, whereas the number and types of sensing units on each sensor are randomly selected. Similarly, the number and types of attributes required to be sensed at each target are all randomly selected as well. Moreover, targets and sensors are randomly deployed in the sensing field. The locations of sensors and targets are fixed during the whole simulation. The sensing range of each sensing unit is the same and is set as 50 m. The communication range of each sensor is twice of the sensing range. A reliable communication channel is assumed in these simulations. All measurements are averaged over 10 runs, if not otherwise specified.

Since the ILP solution is a centralized scheme, no control and computation overheads are counted. Therefore, two different scenarios are considered. In the first scenario, REFS and EEFS are compared with the ILP solution to show the efficiency of the proposed schemes without the control and computation overheads, where the ILP solution is implemented by ILOG CPLEX [24] optimization library. In the second scenario, REFS and EEFS are evaluated when the control and computation overheads are considered. In order to show the effectiveness of the proposed algorithms, a straightforward scheme, named *m*-SU-CTC, is compared in this scenario. As mentioned above, there existed several schemes in the literature considering the CTC problem on a wireless homogeneous sensor network, where each sensor is equipped with only one sensing unit and only one attribute is required to be sensed at each target. Therefore, the *m*-SU-CTC will apply this kind of scheme multiple times, each for the required attribute to be sensed at the targets, so that all the attributes required to be sensed by every target are sensed by the sensors with those specific sensing units on sensors.

Energy consumption and network lifetime are mainly evaluated to verify the performance of the proposed schemes. As mentioned above, the network lifetime is defined as the time interval from the beginning to the time that either the attributes required to be sensed at any target can not be sensed anymore or the sensed data can not be delivered to the sink. On the other hand, the energy consumption models for sensing, communication, and computation are different in scenarios 1 and 2. In scenario 1, the initial energy of each sensor is 50 units. The energy consumption of each type of sensing unit is assumed linearly proportional to the type of the sensing unit. That is, the first type of the sensing unit is assumed to consume one unit of energy in a round, the second type of the sensing unit consumes two units of energy, and likewise. Additionally, the communication module of a sensor consumes one unit of energy to send or receive a unit of the sensed data.

In scenario 2, the energy consumption caused by control and computation overheads are considered. The energy consumption model of MICA2 [4] is adopted in the simulation. In addition, the initial energy of each sensor is assumed 2000 *J*. A sensor in active mode consumes 10.9 *mA*. Similar to scenario 1, the energy consumption of each type of the sensing unit is also assumed linearly proportional to the type of the sensing unit. However, the unit of the energy consumption of the sensing units is *J/min*. If a sensor decides to turn on any sensing unit, the sensor is assumed to reply the sensed data to the sink every 10 minutes. Each round is 100 minutes. Table 3 summaries the energy consumption model used in scenario 2.

Since there is a system parameter ɛ involved in the design of the EEFS in order to obtain an appropriate value of ɛ for scenarios 1 and 2, the experiment contains two parts. The first part is to observe the impact of ɛ on the performance of EEFS for scenarios 1 and 2. According to the results obtained from the first part, the second part of the experiment evaluates the performance of the proposed schemes. As mentioned above, scenario 1 compares the performance of the proposed schemes, REFS and EEFS, against the ILP solution, where the control and computation overheads are not taken into account. In scenario 2, the proposed schemes are compared against a heuristic scheme, where the control and computation overheads are considered.

### 5.1. The Impact of ɛ

In EEFS, ɛ is a system parameter considering the difference of the energy consumption between the communication unit and the sensing units. The use of the ɛ is to make the best use of the sensing and communication units and increase the network lifetime. Therefore, this part of experiment focuses on the impacts of ɛ on the network lifetime for scenarios 1 and 2. In this experiment, three types of attributes are required to be sensed and there are 25 targets and 300 sensors randomly deployed in the 300 m * 300 m sensing field.

**Figure 4.**
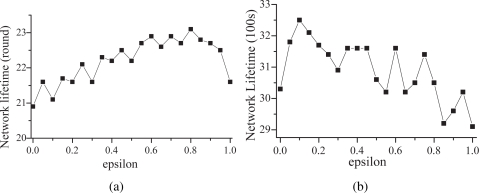
The impact of ɛ on the network lifetime for (a) scenario 1 and (b) scenario 2.

Figures 4(a) and 4(b) show the results for scenarios 1 and 2, respectively. According to Figure 4(a), basically, the network lifetime increases with the increase of ɛ until ɛ = 0.8. The best performance is observed when ɛ = 0.8. In scenario 1, the energy spent in sensing is close to that spent in communication, which implies that the cost of the communication unit is higher than that of the sensing unit. As mentioned above, the sensor should pay more attention on the connectivity contribution. Consequently, ɛ should be set high, which is also coincident with the simulation results shown in Figure 4(a). Note that, in scenario 1, the control overhead is not taken into account. However, if the control overhead is taken into consideration, ɛ should be larger than 0.8.

On the other hand, in scenario 2, the energy cost of the sensing unit is much higher than that of the communication unit. Therefore, the sensor should have a higher tendency toward the coverage contribution. As a result, ɛ should be set low to have the sensor toward the coverage tendency. According to Figure 4(b), the network lifetime decreases with the increase of ɛ. The network obtains the longest lifetime when ɛ = 0.1. Consequently, for the following simulations, ɛ are set to 0.8 and 0.1 for scenarios 1 and 2, respectively.

### 5.2. Performance Evaluations of the Proposed Schemes

#### Energy consumption taken from a snapshot

**Figure 5.**
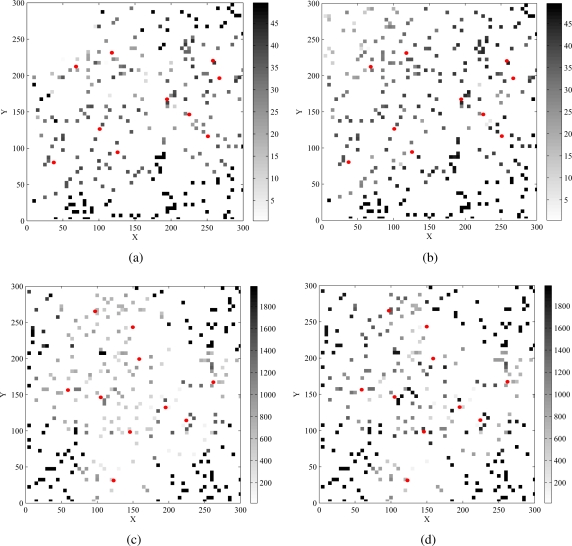
The snapshots of the remaining energy of sensors by (a) REFS and (b) EEFS in scenario 1 and (c) REFS and (d) EEFS in scenario 2, where the snapshots are taken from the end of the 27th round in scenario 1 and are taken from the 3800th minute in scenario 2.

Figure 5 shows the snapshots of the remaining energy of each sensor in the last round of REFS and EEFS, respectively. The circles are the targets to be sensed and the gray squares represent the remaining energy of the corresponding sensor. The darker the gray color of the sensor is, the more remaining energy the sensor has. Figure 5 shows the snapshots of the remaining energy of the sensors in the last round of REFS and the corresponding round of EEFS both in scenarios 1 and 2. On average, the gray squares in Figures 5(b) and 5(d) are darker than those in Figures 5(a) and 5(c), whcih means that the remaining energy of the sensors in EEFS is higher than that in REFS. Moreover, in addition to the remaining energy, the number of remaining sensors (gray squares) in EEFS is also more than that in REFS, especially in scenario 2. These all imply that EEFS performs more efficiently than REFS.

#### Energy consumption and remaining energy in a run

**Figure 6.**
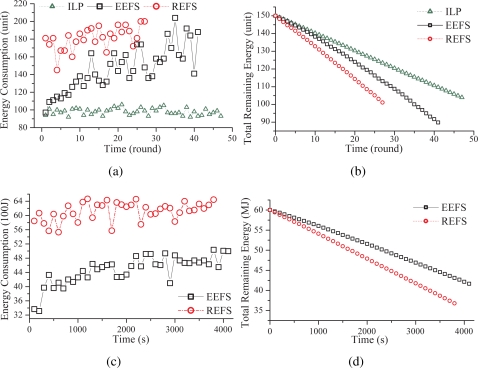
The energy consumption and the remaining energy in each round for the some run. (a) The energy consumption and (b) the remaining energy in each round for some run in scenario 1. (c) The energy consumption and (d) the remaining energy in each round for some run in scenario 2.

To look into the detailed performance of REFS, EEFS, as well as the ILP solution, Figure 6 illustrates the energy consumption and the remaining energy in each round, which is randomly taken from some run, rather than the average of 10 runs. In addition to the settings of the scenarios 1 and 2 described above, the other settings are as follows. The numbers of sensors, targets, and attributes are 300, 10, and 3, respectively.

Figure 6(a) shows the energy cost of REFS, EEFS, and the ILP solution consumed in each round for some run in scenario 1. Obviously, the energy consumptions of REFS and EEFS fluctuate with time, while that of the ILP solution stays stable. This is because the sensing redundancy problem in the ILP solution is slight. Therefore, the energy consumption of the ILP solution in each round is very similar. On the contrary, REFS and EEFS are heuristic. The energy consumptions of REFS and EEFS in each round depend heavily on the number of sensing and communication units selected. Therefore, the energy consumptions of REFS and EEFS have a large variation among rounds. Moreover, since the sensing redundancy problem is much more serious in REFS, the energy consumption of REFS is always the worst and the network lifetime of REFS is the shortest. In addition, in EEFS, sensors consume less energy to perform the sensing tasks at the beginning because many redundant sensing units are removed. However, when the number of failed sensors increases, some sensing responsibilities of sensors may become the critical sensing responsibilities. Therefore, the sensing redundancy problem will be getting worse, which will result in a higher energy consumption of the sensors in a round.

On the other hand, although the energy consumption of EEFS is higher than that of the ILP solution in each round, the network lifetime of EEFS is still very close to that of the ILP solution, whose difference is smaller than eight rounds, as shown in Figure 6(b). Figures 6(c) and 6(d) illustrate the energy consumption and the remaining energy of REFS and EEFS in each round when the control and computation overheads are considered. The results are similar to those shown in Figures 6(a) and 6(b).

#### The impacts of the numbers of sensors, targets, and attributes on network lifetime

**Figure 7.**
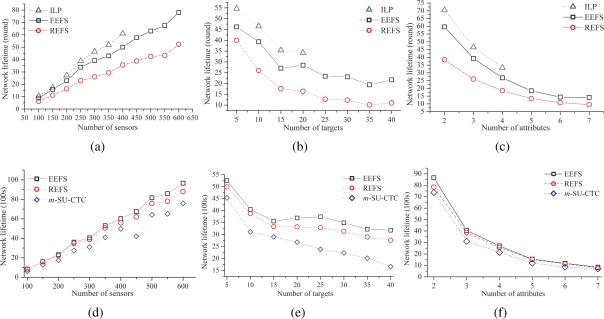
The impacts of the numbers of (a) sensors, (b) targets, and (c) attributes in scenario1 as well as (d) sensors, (e) targets, and (f) attributes in scenario 2 on the network lifetime.

The following experiments are to observe the impacts of *the number of sensors*, in which the number of targets and the number of attributes are fixed at 10 and 3, *the number of targets*, in which the number of sensors and the number of attributes are fixed at 300 and 3, and *the number of attributes*, in which the number of sensors and the number of targets are fixed at 300 and 10, on the network lifetime with regard to REFS, EEFS, and the ILP solution. The results for scenarios 1 and 2 are shown in Figure 7.

Figures 7(a), 7(b) and 7 (c) illustrate the simulation results of scenario 1. Since the ILP solution becomes extraordinarily complicated when the numbers of attributes, targets or sensors increase, some results of the ILP solution are unavailable. Obviously, the network lifetime increases with the increase of the number of sensors. On the contrary, when the number of targets or attributes increases, the network lifetime decreases accordingly. However, the ILP solution always has the longest network lifetime, if available, whereas REFS is the worst in all cases. Nevertheless, the performance of EEFS is close to that of the ILP solution. From the practical viewpoint, EEFS is a promising alternative solution.

Figures 7(d), 7(e) and 7(f) respectively show the impacts of the number of sensors, targets, and attributes on network lifetime when the control and computation overheads are taken into consideration in scenario 2. Here, the communication overhead takes the messages exchanged among sensors into account, such as the sensor’s capabilities, decision announcement, etc.

Basically, Figures 7(d), 7(e) and 7(f) are similar to Figures 7(a), 7(b) and 7(c), respectively. As indicated in Figure 7(d), EEFS, REFS, and *m*-SU-CTC extend the network lifetime when the number of sensors increases. Among them, EEFS performs the best when a large number of sensors are deployed in the area of interest, even though EEFS needs extra control and computation overheads.

Figure 7(e) illustrates the impact of the number of targets on the network lifetime. It can be found that the network lifetime of *m*-SU-CTC rapidly decreases when the number of targets increases. However, the network lifetimes of EEFS and REFS decrease smoothly. That is because, with the aid of the proposed algorithms, the increase of the number of targets does not activate too many sensing units on sensors. The simulation result also indicates that *m*-SU-CTC is unsuitable for WHSNs when a large number of targets needs to be sensed.

According to Figure 7(f), the impact of the number of attributes on the network lifetime is not significant when the number of attributes is larger than 5. Basically, EEFS performs better than REFS and *m*-SU-CTC. However, the differences of the network lifetime among EEFS, REFS and *m*-SU-CTC are not significant, especially when the number of attributes is larger than 5. Since only 300 sensors are deployed in the area of interest and many kinds of attributes of the targets are needed to be sensed, a lot of sensors have to be activated to sense the required attributes. As a result, the effect of the policies to remove redundant or inefficient sensing responsibilities in the EEFS scheme is not obvious. Thus, the differences of the network lifetime between EEFS and REFS are not significant. The result is consistent with that in Figure 7(d), which indicates that the number of sensors to be deployed should be increased when the number of attributes increases.

It is worth noticing that *m*-SU-CTC performs the worst in all cases. It is because that *m*-SU-CTC is originally designed for the sensor equipped with only one sensing unit, which is unsuitable to be applied for WHSNs. Without good coordination among sensing units or sensors, *m*-SU-CTC will turn on many redundant sensing units or sensors, and thus, result in higher energy consumption.

#### Comprehensive comparisons of REFS and EEFS

**Figure 8.**
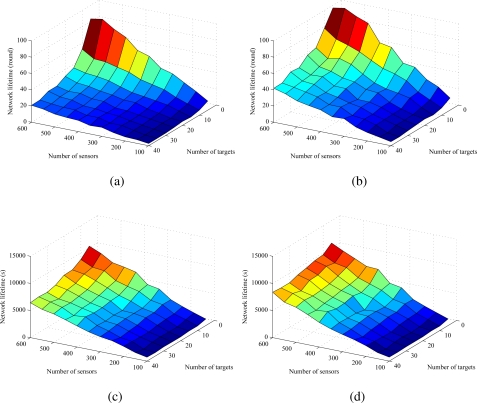
Comprehensive comparisons of REFS and EEFS. The impact of the numbers of sensors and targets on the network lifetime in terms of (a) REFS and (b) EEFS in scenario 1 as well as (c) REFS and (d) EEFS in scenario 2.

Figure 8 shows the performance of REFS and EEFS in a comprehensive view combining the numbers of sensors and targets to observe their impact on the network lifetime. In the simulation, the number of sensors is varied from 100 to 600 with a step of 100, the number of targets is varied from 5 to 40 with a step of 5, and the number of attributes is fixed at 3.

In scenario 1, both REFS and EEFS have similar inclinations that the network lifetime increases with the increase of the number of sensors, but decreases with the increase of the number of targets, as shown in Figures 8(a) and 8(b). However, EEFS still shows a better performance than REFS. When the control and computation overheads are taken into consideration, both REFS and EEFS also have similar inclination that the network lifetime increases with the increase of the number of sensors, but decreases with the increase of the number of targets, as shown in Figures 8(c) and 8(d). Also, EEFS has better performance than REFS. In addition, when control and computation overheads are considered, the impact of the number of targets on the network lifetime is not significant. That is because when the number of targets increases, the energy consumption in sensing the required attributes does not increase substantially. It is shown again that, in practical view, when multiple attributes are required to be sensed, deploying a WHSN with multiple sensing units is a promising solution.

### 5.3. Summary

The simulation results can be summarized as follows.

1. The network lifetime increases with the increase of the number of sensors.
2. The network lifetime decreases with the increase of the number of targets.
3. The network lifetime decreases with the increase of the number of attributes.
4. The performance of EEFS is very close to that of the ILP solution, which is an optimal solution.
5. According to the simulation results, even though the control overhead of EEFS is higher than that of REFS, the performance of EEFS is still better than that of REFS. However, since the computation cost of EEFS is higher than that of REFS, if the computation capability of a sensor is not good enough, REFS may be a better choice.
6. The modified algorithm *m*-SU-CTC originally designed for the sensor with one sensing unit may not work well in WHSNs because *m*-SU-CTC does not jointly take the sensing abilities of the equipped sensing units into consideration.
7. Even though the ILP solution performs the best, REFS and EEFS are still the practical solutions to WHSNs.

## 6. Conclusions

Coverage and connectivity are important measurements for the quality of surveillance that a sensor network can provide. Therefore, the paper emphasizes on the connected target coverage problem in wireless heterogeneous sensor networks with multiple sensing units, termed the MU-CTC (Multiple sensing **U**nits for **C**onnected **T**arget **C**overage) problem. The problem is to schedule the activity of each sensing unit on each sensor to completely cover the targets of interest, to make sensors relay the sensed data to the sink, and, subject to the energy constraint of each sensor, to maximize the network lifetime. The problem is further reduced to a connected set cover problem, called the MU-CSC (**M**ultiple sensing **U**nits for **C**onnected **S**et **C**over) problem. According to the MU-CSC problem, several ILP constraints are proposed. In addition, two distributed schemes, REFS and EEFS, are proposed to solve the MU-CTC problem. These two schemes are executed by each sensor in the initial phase of each round. In REFS, each sensor enables its sensing units by its remaining energy and neighbors’ decisions. However, in EEFS, the coverage and connectivity contributions are also taken into account. Simulation results show that REFS and EEFS can prolong the network lifetime effectively. The performances of the two schemes are close to that of the ILP solution. However, the ILP solution is a centralized and computationally intensive scheme, so the proposed distributed schemes are much more practical. In addition, the schemes can be easily implemented in a real network.

In the future, different levels of target coverage requirements (e.g., *k*-coverage) will also be considered to meet the sensing requirements of different applications. From the practical viewpoint, the sensing units with different sensing ranges will also be taken into consideration in the future.

## Figures and Tables

**Table 1.** Related work.

Scheme	TC / CTC	Distributed	Sensing Unit
[13]	TC	No	Single
[14]	TC	Yes	Single
[15]	TC	No	Single
[16]	CTC	No	Single
[17]	CTC	No	Single
[18]	CTC	No	Single
[19]	CTC	Yes	Single
[20]	CTC	Yes	Single
[21]	TC	Yes	Multiple

**Table 2.** Simulation setting for MU-CTC problem.

Sensing field	300 m × 300 m
Sensing range	50 m
Communication range	100 m
Number of sensors	100 .. 600
Number of targets	5 .. 40
Number of attributes	2 .. 7

**Table 3.** Energy consumption model for MU-CTC problem when control and computation overheads are considered.

Active	10.9 *mA*				
Transmit (+4 *dBm*)	11.6 *mA*				
Receive	7.0 *mA*				

Sensing unit	*u*^1^	*u*^2^	*u*^3^	…	*u^l^*
Energy consumption (*J/min*)	1	2	3	…	*l*
